# Mathematical Modeling and Dynamic Simulation of Metabolic Reaction Systems Using Metabolome Time Series Data

**DOI:** 10.3389/fmolb.2016.00015

**Published:** 2016-05-03

**Authors:** Kansuporn Sriyudthsak, Fumihide Shiraishi, Masami Yokota Hirai

**Affiliations:** ^1^RIKEN Center for Sustainable Resource ScienceYokohama, Japan; ^2^Department of Bioscience and Biotechnology, Graduate School of Bioresource and Bioenvironmental Science, Kyushu UniversityFukuoka, Japan

**Keywords:** biochemical systems theory, bottleneck ranking indicator, dynamic simulation, mathematical model, metabolic reaction network, metabolome, sensitivity analysis, time series data

## Abstract

The high-throughput acquisition of metabolome data is greatly anticipated for the complete understanding of cellular metabolism in living organisms. A variety of analytical technologies have been developed to acquire large-scale metabolic profiles under different biological or environmental conditions. Time series data are useful for predicting the most likely metabolic pathways because they provide important information regarding the accumulation of metabolites, which implies causal relationships in the metabolic reaction network. Considerable effort has been undertaken to utilize these data for constructing a mathematical model merging system properties and quantitatively characterizing a whole metabolic system *in toto*. However, there are technical difficulties between benchmarking the provision and utilization of data. Although, hundreds of metabolites can be measured, which provide information on the metabolic reaction system, simultaneous measurement of thousands of metabolites is still challenging. In addition, it is nontrivial to logically predict the dynamic behaviors of unmeasurable metabolite concentrations without sufficient information on the metabolic reaction network. Yet, consolidating the advantages of advancements in both metabolomics and mathematical modeling remain to be accomplished. This review outlines the conceptual basis of and recent advances in technologies in both the research fields. It also highlights the potential for constructing a large-scale mathematical model by estimating model parameters from time series metabolome data in order to comprehensively understand metabolism at the systems level.

## Introduction

Systems biology has become an important research field to fully understand the complex metabolism of cells in living organisms *in toto* (Kitano, 2002a,b; Aderem, 2005; Kirschner, 2005). It involves numerous techniques including -omics to systematically identify, analyze, control, and design metabolic systems (Ukai and Ueda, 2010) at gene, transcript, protein, and metabolite levels. Among the -omics, metabolomics is one of the newest -omics sciences (Rochfort, 2005; Preidis and Hotez, 2015). The metabolome, which is affected by changes in the transcriptome and proteome, is considered to be downstream in the layer of multi-omics. It is also most directly related to the visible phenotype of biological systems. A complete understanding of metabolism through use of metabolome data is greatly anticipated. This involves the development of high-throughput analytical instruments to provide a wide range of metabolic profiles along with the improvement of data processing techniques to accurately identify or annotate metabolites from mass spectra and precisely measure their quantities in a living cell (Fiehn, 2002; Weckwerth, 2003; Bain et al., 2009). This information can then be implemented to advance intuitive and functional concepts for designing and engineering ideal metabolic systems.

An assortment of statistical methods have been developed and utilized to identify correlations or differences among metabolome data of biological samples. Nevertheless, novel strategies are required to systematically elucidate the regulatory mechanisms of metabolites in greater detail. Time series metabolome data is suited for this task. Time series data of the response of a metabolic system to internal and/or external stimuli contain important information of different aspects of the dynamic characteristics (Voit, 2013) of the metabolic system regardless of the type of organism. For example, time series metabolic data were used to elucidate regulatory mechanisms of respiratory oscillations of yeast (Murray et al., 2007) and their adaptation to temperature stress (Strassburg et al., 2010). Time series metabolome data were also used to understand adaptation mechanisms of plants to cold (Espinoza et al., 2010), salinity (Kim et al., 2007), dehydration (Urano et al., 2008), and thermal stress such as to heat- and cold-shocks (Kaplan et al., 2004). In humans, time series metabolome data were collected to determine metabolic profile responses of stored red blood cells to hypoxia (Kinoshita et al., 2007), which included monitoring ATP levels, as well as to validate mathematical models of dynamic characteristics and behaviors of erythrocyte metabolism (Nishino et al., 2013). Time series data is not only useful for validating simulated metabolic behaviors predicted from a mathematical model generated using enzyme kinetics, but these data are also a powerful component for directly generating a kinetic model to systematically understand metabolic behaviors and characteristics of a metabolism of interest. Kinetic models can be used to further predict possible unknown regulatory mechanisms controlling a metabolic pathway.

**Figure 1:**
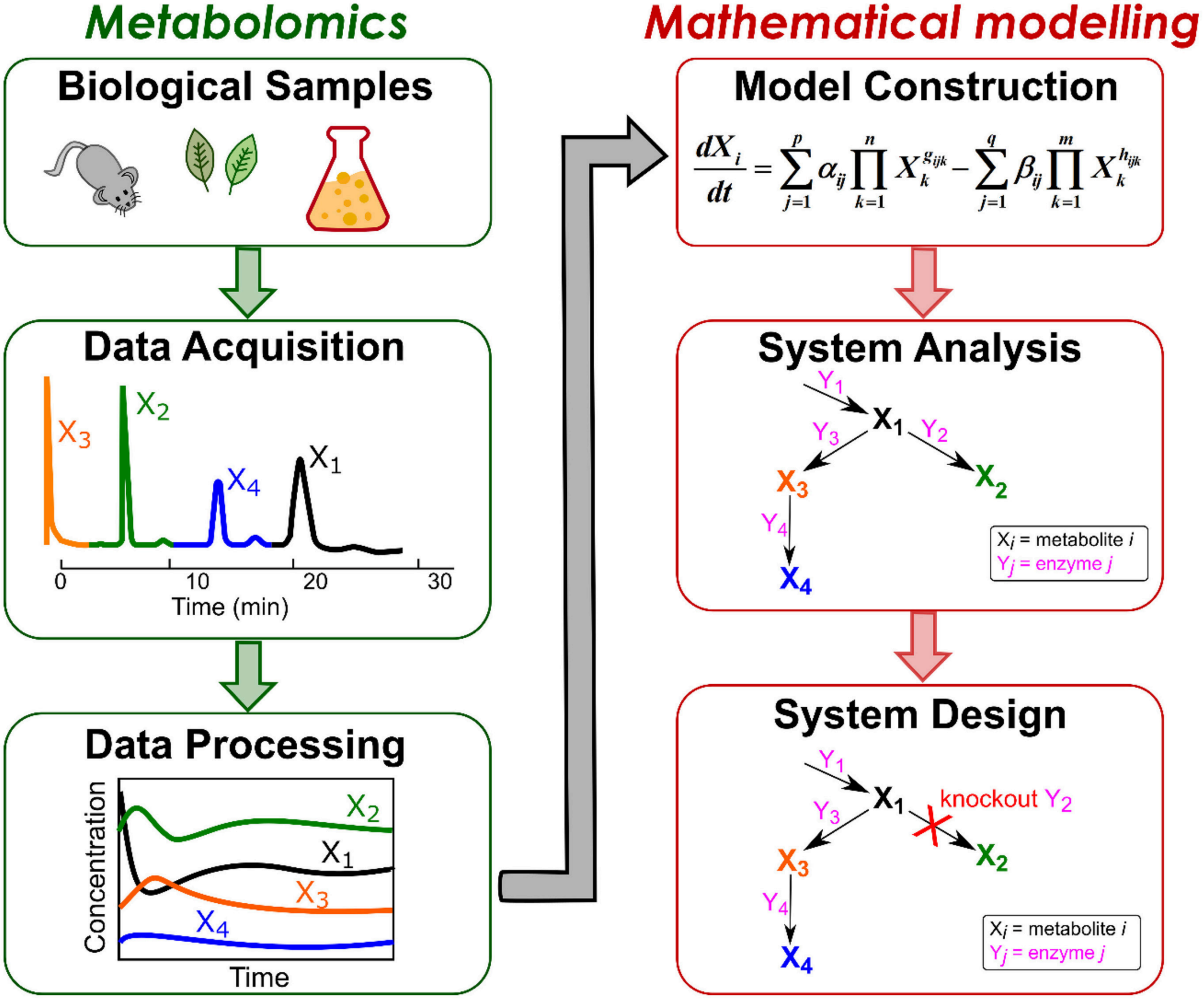
**Outline for mathematical modeling using metabolome data**. The workflow includes metabolomics approaches for acquiring and processing metabolic data from biological samples as well as mathematical approaches for constructing and analyzing mathematical model to design an optimal system.

An overview of mathematical modeling using metabolome data is illustrated in Figure 1. In general, the workflow begins with sampling biological data consisting of simultaneously-acquired metabolic profiles. The profiles are processed and utilized for constructing a mathematical model, which can be exploited to analyze metabolic systems as well as to design an optimal system of interest. In this review, we address the basic concepts of advancements in both metabolomics and mathematical modeling. We review the methods for metabolome analyses and nature of metabolome data (Section Nature of Metabolome Data) as well as current mathematical modeling approaches (Section Current Status of Modeling Metabolic Reaction Networks), and then describe the procedures to construct a kinetic model from those data (Section Kinetic Models from Time Series Metabolome Data) and use it for system analysis (Section Systems Analysis). Finally, we pinpoint the potential of combining mathematical techniques to construct a large-scale dynamic model for further understanding of biological systems.

## Nature of metabolome data

A living cell contains thousands of metabolites, which differ greatly in their chemical property and abundance (Luca and Pierre, 2000; Griffin and Shockcor, 2004; Prescher and Bertozzi, 2005); therefore, no single analytical instrument is suitable to measure all metabolites (Saito and Matsuda, 2010; Weckwerth, 2011). A combination of different high-throughput instruments, including nuclear magnetic resonance (NMR) spectroscopy, and gas chromatography (GC), liquid chromatography (LC), or capillary electrophoresis (CE) coupled to mass spectrometry (MS), is required to analyze a whole metabolome (Villas-Boas et al., 2005; Pan and Raftery, 2007).

In most cases, analytical procedures to generate metabolome data start with sample preparation, which includes quenching and metabolite extraction (Fukushima and Kusano, 2013). Sample preparation methods are important because they have an effect on the coverage and content of metabolites that can be analyzed (Vuckovic, 2012). Depending on the metabolites-of-interest and instruments used, sample preparation may include derivatization to chemically modify compounds to produce derivatives which have properties suitable for analysis. The spectra and intensities of metabolites are then acquired using suitable instruments. These data are pre-processed to determine the quantity and identity of metabolites. Core technologies for detection can be classified into two types: non-targeted and targeted methods for top-down and bottom-up approaches, respectively (Bain et al., 2009; Hiller et al., 2009).

Non-targeted methods generally aim to identify as many metabolites as possible in a biological sample at once. The identification of metabolites involves instruments (Dunn et al., 2012; Fuhrer and Zamboni, 2015; Sèvin et al., 2015) to detect spectra, i.e., patterns representing the distribution of ions by mass-to-charge ratio (m/z), across wide coverage ranges. Spectra are subsequently pre-processed for base-line correction, peak detection, and peak alignment (Lommen, 2009; Lommen and Kools, 2012) to appropriately identify peaks and mass spectra (Tsugawa et al., 2015) as well as annotate metabolites (Matsuda et al., 2009; Horai et al., 2010). Although non-targeted methods have an advantage in covering a greater percentage of the metabolome, they usually provide only qualitative data. Accordingly, the application of data from non-targeted methods is usually subject to statistical analysis to normalize data by the original mass of the sample and/or by an internal standard to reduce the influence of experimental factors on the data set (Summer et al., 2007). These data can be used to investigate correlations among metabolite intensities due to different environmental conditions (Wechwerth et al., 2004), the existence of metabolites in mutants compared to wide-type specimens (Jonsson et al., 2005; Kim et al., 2015), and for multivariate comparative analysis of metabolic phenotypes (Tikunov et al., 2005).

In contrast to non-targeted methods, targeted methods detect specific metabolites (Badawy et al., 2008; Sawada et al., 2009; Kato et al., 2012); however, targeted methods can be applied to analyze hundreds of metabolites in a semi-quantitative or quantitative manner (Shulaev, 2006). Targeted methods provide quantitative data in absolute concentrations typically by using stable isotope-labeled standard compounds (Birkemeyer et al., 2005). Isotope labeling also permits the quantification of cell fluxomes through metabolic flux analysis (You et al., 2014). Both absolute metabolite concentrations and rates of flux are potential components for building a large-scale mathematical model, which can reveal dynamic associations of metabolites in an entire metabolic system. However, isotope labeling-assisted metabolomics is costly and isotope-labeled compounds are limited. Consequently, most studies, wherein large-scale targeted metabolomics were performed, report metabolic abundances in relative concentrations, or peak intensities rather than in absolute concentrations. Although, both non-targeted and targeted analyses can be used to determine a large number of metabolic profiles at once, it does not necessarily indicate the detection and measurement of all metabolites in a particular metabolic pathway. This presents a challenge for constructing precise mathematical models, which typically require absolute concentrations of all metabolites in a specific pathway.

## Current status of modeling metabolic reaction networks

Mathematical models permit the investigation of metabolic characteristics and behaviors, including of regulatory mechanisms and responses to internal and external environments, to comprehensively understand metabolism. Table 1 tabulates methods commonly used for mathematical modeling. Model types vary from large-scale qualitative models, such as topological and stoichiometric models, to small-scale quantitative models, such as kinetic models (Hartmann and Schreiber, 2015; Novere, 2015). As the stoichiometric and kinetic models seem to have a potential to handle large-scale dynamic systems, they will be discussed in more details.

**Table 1:** **Currently common methods for mathematical modeling**.

**Model types/Theories**	**Fundamental Equations**	**Applications**	**Limitations**
Topological models/Centrality (Ma and Zeng, 2003)	$C\left(x\right)\;=\;\frac{n-1}{\sum y\in U,y\neq x^{d\left(x,y\right)}}$ *C*(*x*), closeness centrality of node *x*; *d*(*x*,*y*), distance between node *x* and node *y*; *U*, set of all nodes; *n*, node number in the network	- Large-scale qualitative model	- Only topological information - No dynamic properties
Stoichiometric models/Flux balance analysis (Palsson, 2015)	*S*·***v*** = 0 *S*, stoichiometric matrix; **v**, vector of fluxes	- Large-scale model with quantitative prediction - Elementary flux modes as alternative (Schuster et al., 1999)	- Steady-state assumption - No dynamic properties
Petri net models (Baldan et al., 2010)	*N* = (*P, T, F*) *N*, elementary net; *P*, places; *T*, transitions; *F*, flow relations	- Qualitative and quantitative information with quantitative predictions and dynamic behavior	- Poor knowledge of kinetic parameters
Kinetic models/Mass action kinetics (Horn and Jackson, 1972)	$r_{\text{f}}=\overset{m}{\underset{i=1}{\sum }}k_{i}A_{i}$ *r*_*f*_, rate equations; *k_*i*_*, molecularities of *A_*i*_*; *A_*i*_*, reactants or metabolite concentrations	- Detailed quantitative description with quantitative predictions and dynamic behavior - Simple formulations	- Small to medium-scales - Lack of information on regulatory mechanisms
Kinetic models/Michalis-Menten kinetics (Bajzer and Strehler, 2012)	$v=\frac{V_{\max}S}{K_{\text{M}}+S}$ *v*, reaction rate; *S*, substrate concentration; *V*_max_, maximum rate achieved by the system; *K*_M_, substrate concentration at which the reaction rate is half of *V*_max_	- Detailed quantitative description with quantitative predictions and dynamic behavior - Parameters directly taken from experiments	- Small-scale model - Complicated equations - Requirement of information on kinetic parameters in details
Kinetic models/Lin-log model (Wu et al., 2004)	$\frac{v_{i}}{J_{i}^{0}}=\frac{e_{i}}{e_{i}^{0}}\left(1+\overset{n+m}{\underset{j=1}{\sum }}\varepsilon_{ij}^{0}\ln\;\left(\frac{X_{j}}{X_{j}^{0}}\right)\right)$ *X_*j*_*, metabolite concentrations; *v_*i*_*, reactions; $J_{i}^{0}$, fluxes; $\varepsilon_{ij}^{0}$, reference elasticity; *e*_*j*_, reference level of enzyme activity	- Detailed quantitative description with quantitative predictions and dynamic behavior - Related to metabolic control analysis	- Small to medium-scale model - Requirement of approximated kinetic parameters - Systems close to steady-state
Kinetic models/Metabolic control analysis (Kacser and Burns, 1973; Heinrich and Rapoport, 1974)	$C_{ij}^{J0}=\frac{e_{j}^{0}}{J_{i}^{0}}\cdot {\left(\frac{dJ_{i}}{de_{i}}\right)}^{0}$; $\varepsilon_{ik}^{x0}=\frac{x_{k}^{0}}{v_{i}^{0}}\cdot {\left(\frac{\partial v_{i}}{\partial x_{k}}\right)}^{0}$ *C*^*J*^, flux control coefficient; *J*_*i*_, fluxes; *e*_*j*_, enzyme level around reference (quasi) steady-state; ε^*x*^, elasticities; *x*_*k*_, metabolite concentrations; *v*_*i*_, reaction rates	- Detailed quantitative description with quantitative predictions and dynamic behavior - Quantification of changes in metabolic variables including fluxes and concentrations	- Small to medium-scale model - Requirement of approximated kinetic parameters - Systems close to steady-state
Kinetic models/Biochemical systems theory (Savageau, 1969)	S-system: $\frac{dX_{i}}{dt}=\alpha_{i}\prod_{j=1}^{n}X_{j}^{g_{ij}}-\beta_{i}\prod_{j=1}^{m}X_{j}^{h_{ij}}$ GMA-system: $\frac{dX_{i}}{dt}=\overset{p}{\underset{j=1}{\sum }}\alpha_{ij}\prod_{k=1}^{n}X_{k}^{g_{ijk}}-\;\overset{q}{\underset{j=1}{\sum }}\beta_{ij}\prod_{k=1}^{m}X_{k}^{h_{ijk}}$ *X*_*i*_, metabolite concentrations; α_*i*_, β_*i*_, rate constants of total influxes and effluxes;α_*ij*_, β_*ij*_, rate constants of influxes and effluxes; *g*_*ij*_ (*g*_*ijk*_), *h*_*ij*_ (*h*_*ijk*_), kinetic orders of influxes and effluxes	- Detailed quantitative description with quantitative predictions and dynamic behavior - Less number of parameters comparing to Michalis-Menten kinetics - Simplification	- Small to medium-scale model - Requirement of approximated kinetic parameters

Stoichiometric modeling typically uses a static model based on the assumption of steady-state; therefore, it does not require kinetic information and can handle a large-scale system. A well-known stoichiometric model is genome-scale metabolic reconstruction using flux balance analysis (Orth et al., 2010; Palsson, 2015). It is a constraint-based model using linear programming to optimize metabolic fluxes based on stoichiometric coefficients of each reaction throughout the entire metabolic network; it principally requires only steady-state absolute concentrations for optimization. Genome-scale metabolic reconstruction is straightforward and can be used for the prediction of cellular phenotypes, analysis of biological network properties, as well as metabolic engineering (McCloskey et al., 2013). Thus, it has been applied in the analysis of various organisms, including *Escherichia coli* (Edwards and Palsson, 2000; Orth et al., 2011), *Saccharomyces cerevisiae* (Herrgård et al., 2008), *Arabiodopsis thaliana* (Poolman et al., 2009; de Oliveira Dal'Molin et al., 2010; Mintz-Oron et al., 2012), and humans (Thiele et al., 2013).

In contrast, kinetic modeling, which generates a dynamic model, requires kinetic rate equations, and model parameters to provide detailed quantitative description, quantitative prediction and dynamic behaviors. These requirements make kinetic modeling more complicated and limit its application to only small-scale systems. Dynamic models can be classified into two classes: stochastic and deterministic (Puchalka and Kierzek, 2004; Shmulevich and Aitchison, 2009). Stochastic or probabilistic models incorporate randomness or uncertainty to characterize the performance of a system, and use values generated from probability distributions rather than a fixed unique value. In contrast, deterministic models capture the collective behaviors of the elements constituting the network. Again, they require a set of variable states uniquely determined by parameters using optimization methods and experimental data.

Recent efforts have been made to implement dynamic characteristics into genome-scale reconstruction models in order to consider the entire dynamic system. The common approach to observe dynamic characteristics is to use dynamic flux balance analysis (dFBA). For example, dFBA has been used to link a Monod kinetic model and genome-scale flux balance analysis to analyze the dynamic metabolism of environmentally important bacterium (Feng et al., 2012). Although this method cannot offer a fully dynamic state, it can still provide pseudo-dynamic characteristics to predict directions of metabolic fluxes with changes in gene expression or enzyme activities. Alternatively, genome-scale metabolic reconstruction models can also be integrated with *in vivo* metabolome data via the differential biochemical Jacobian (Nägele et al., 2014) to define a metabolic interaction matrix. The differential Jacobian is calculated using a metabolic reaction matrix and covariance of metabolome data. It combines dynamic modeling strategies with large-scale steady state profiling approaches without explicit knowledge of individual kinetic parameters. The Jacobian might permit design parameter optimization strategies for ODE-based kinetic models of metabolic systems in the near future.

## Kinetic models from time series metabolome data

Kinetic models comprise two components: symbolic formulas of model equations and numerical values of model parameters. Most model equations are expressed as ordinary differential equations (ODEs) of various forms (Table 1), such as linear approximations, enzyme kinetic rate laws using Michaelis-Menten kinetics (Bajzer and Strehler, 2012), linear-logarithmic approximations (Hatzimanikatis and Bailey, 1996; Visser and Heijnen, 2002), and power-law equations based on metabolic control analysis (MCA; Kacser and Burns, 1973; Heinrich and Rapoport, 1974) or biochemical systems theory (BST; Savageau, 1969; Voit, 2000). Regardless of the chosen format of model equations, numerical values determining metabolite concentrations and reaction rates are required. This involves inverse problems using experimental data (or results) to calculate model parameters (or causes). Since experimental data contain not only a wealth of information but also biological variation and analytical errors, parameter estimation from actual biological data remains a challenging task, which usually represents a bottleneck in the modeling process (Voit, 2012).

### Parameter estimation

Typical approaches for estimating parameter values include determining enzymatic kinetic rates through *in vitro* enzymatic assays (bottom-up) or indirectly estimating from metabolic time series data (top-down). The bottom-up method is a conventional method in which each model parameter is experimentally determined and then integrated into a final model. It has been applied to several organisms; however, it requires considerable time and financial support to conduct experiments to obtain parameter values of every individual reaction.

As an alternative and with the aim of constructing kinetic models using limited data sets and requiring less time, theoretical researchers have proposed new algorithms for simultaneously estimating model parameters using only time series data of metabolite concentrations. In general, parameter values are determined by minimizing an objective function measuring the difference between experimental data (time series data) and model predictions (predicted data). Various optimization algorithms to find global or local optima were previously reviewed in detail (Mendes and Kell, 1998; Chou and Voit, 2009). Standard optimization methods for global optimization in metabolic modeling include genetic algorithms (Michalewicz, 1994), evolutionary programming (Fogel et al., 1992), and simulated annealing (Corana et al., 1987), whereas those for local optimization include Newton-Raphson (Press et al., 2002, 2007) for linear least-squares analysis and Levenberg-Marquardt (Levenberg, 1944; Marquardt, 1963; Gavin, 2011) for non-linear least-squares analysis. Both global and local optimization algorithms have their advantages and disadvantages. In short, most global optimization algorithms search for the global minimum across all inputs, which require a set of functions, boundaries, constraints, and high computational costs. Local optimization algorithms search only for the local minimum but are computationally less expensive. Some researchers have even reduced these constraints to the no free lunch theorem in search and optimization (Wolpert and MacReady, 1997), claiming that there is no perfect algorithm that can guarantee a solution in a reasonable time and space unless the model equations and experimental data are optimally suited to solve a problem. Consequently, although various tools for kinetic modeling have been used (Alves et al., 2006), selecting an optimization method still depends on data characteristics, types of model equations, or even the preferences and experiences of the modeler.

The fewer the number of model parameters, parameter estimation becomes simpler and more accurate. Several research groups have been working on the development of mathematical approaches to reduce the number of parameters in parameter estimation by taking advantage of the simplification of S-system formulation (approximate power law representation) within biochemical systems theory (BST). Numerous approaches to estimate parameter values using S-system formulation together with time series data of metabolic concentrations have been proposed. This includes alternative regression (Chou, 2006), automated procedure (Marino and Eberhard, 2006), Newton flow (Kutalik et al., 2007), automated smoother for decoupling (Vilela et al., 2007), neutral (Vilela et al., 2009), two-phase dynamic (Jia et al., 2011), estimation of dynamic flux profiles (Chou and Voit, 2012), Newton-Raphson (Iwata et al., 2014), and PENDISC method (Sriyudthsak et al., 2014a). In addition, approximate estimation methods for large-scale analysis include coarse (Iwata et al., 2013) and U-system (Sriyudthsak et al., 2014b) approaches, which were proposed for predicting coarse metabolic parameters, including those of unmeasurable metabolites.

In general, these optimization algorithms perform well with training data generated *in silico* with low noise. Unfortunately, biological data is characterized by high variance from both biological variation and analytical errors. In addition, larger-sized models involve the estimation of more parameters. The majority of metabolome data are also reported in relative concentration units, which sometimes result in problems when balancing the absolute amounts and stoichiometry of parameter values. Ultimately, a useful model must be able to reproduce biological observations before being used to predict different biological scenarios. Thus, a reasonable number of model equations and model parameters are required, even though a compromise between model accuracy and model size remains a challenge.

## Systems analysis

Once a given metabolic reaction system has been described using a mathematical model, systems analysis can be performed to characterize the following characteristics of the reaction network.

### Eigenvalues

The stability and oscillatory behavior of a system is usually identified by means of eigenvalues, which are originally a special set of scalars associated with a linear system of equations. The same concept can be employed for nonlinear systems expressed by differential equations describing the time rate of change in metabolite concentrations. In this case, a steady state is commonly used as an operating point to approximately linearize the systems. The eigenvalues are the roots of the characteristic equations derived from the differential equations under a steady state assumption. In general, *n* differential equations for metabolite concentrations provide the same number of eigenvalues. The eigenvalue is generally a complex number with real and imaginary parts, which can reveal at least four different network behaviors. First, if all the real parts are negative, the nominal steady state is locally stable and the system will return to steady state following small perturbations. Second, if at least one imaginary part is nonzero, metabolite concentrations may oscillate under certain conditions. Third, if the absolute value of the ratio of maximum to minimum values of the real parts, i.e., stiffness ratio, is very large, metabolite concentrations vary at significantly different rates, and some may require very long times to reach steady state (Shiraishi and Savageau, 1992c). For example, if the stiffness ratio is >10^4^, the system is judged to be stiff (Shiraishi and Savageau, 1992a), which makes it difficult to numerically solve relevant differential equations. Fourth, the absolute value of the minimum value of the real parts can be approximately regarded as the rate constant of a first-order reaction and therefore can be expressed in units of time. Consequently, the reciprocal of the value provides an approximate time at which all metabolite concentrations return to the previous steady state following perturbations (it would take about three-folds of the estimated time until all metabolite concentrations sufficiently return to steady state).

### Steady state sensitivity

The sensitivity of a reaction network at steady state is an essential measure to characterize the reaction network. BST defines several kinds of sensitivities (logarithmic gain, rate-constant sensitivity, and kinetic-order sensitivity) (Shiraishi and Savageau, 1992a,b,c,^1993^; Savageau, 2009). The most important and commonly used sensitivity value is the logarithmic gain, which expresses the percentage change of a dependent variable (metabolite concentration *X*_*i*_ and flux *V*_*i*_) in response to the infinitesimal percentage change of an independent variable (enzyme activity *Y*_*j*_). The equations for metabolite concentration and flux are:

(1) $$\begin{array}{l} L\left(X_{i},Y_{j}\right)={\left(\frac{\partial X_{i}}{\partial Y_{j}}\right)}^{*}\frac{Y_{j}^{*}}{X_{i}^{*}},L\left(V_{i},Y_{j}\right)={\left(\frac{\partial V_{i}}{\partial Y_{j}}\right)}^{*}\frac{Y_{j}^{*}}{V_{i}^{*}} \end{array}$$

where, the ^*^ symbol indicates that results are evaluated at the nominal steady state. A larger absolute logarithmic gain value indicates that the dependent variable is more strongly affected by the independent variable. A positive value of *L*(*X*_*i*_,*Y*_*j*_) indicates that the dependent variable (metabolite concentration) at a new steady state achieved by a change in an independent variable (enzyme activity) takes a value larger than that at the previous steady state, whereas a negative value indicates the opposite response. The software, COSMOS (Shiraishi et al., 2014), performs calculations of steady-state metabolite concentrations, transformations of differential equations with various types of flux expressions into those with power-law flux expressions in S-system or GMA-system form (Table 1), and calculations of logarithmic gains and eigenvalues with high accuracy. It achieves high accuracy by implementing the complex-step method (Lyness and Moler, 1967), by which numerical derivatives can be calculated to 14–16 significant digits of accuracy in double precision. Thus, all calculated values are reliable even when the number of differential equations is very large. Moreover, COSMOS can find steady-state metabolite concentrations accurately, even for a large-scale system, by first numerically solving differential equations until metabolite concentrations do not change remarkably and then using those values as initial estimates in root-finding iterations.

### Dynamic sensitivity

When there is no steady state or when there exists a steady state but the objective is to observe and analyze the dynamics of a system, metabolite concentrations that vary with time must be considered. In these cases, steady state sensitivities can no longer be calculated. Hence, time-varying sensitivities, i.e., dynamic sensitivities (dynamic logarithmic gains), must be calculated (Shiraishi et al., 2005, 2009; Sriyudthsak et al., 2015). The first step of this calculation is to partially differentiate the differential equations for metabolite concentrations with an independent or dependent variable of interest in order to obtain the differential equations for sensitivities, i.e., sensitivity equations (Dickinson and Gelinas, 1976). This mathematical operation is laborious and mistakes are trivial to make especially when the system is large. This problem can be overcome by utilizing the function of symbolic differentiation in the commercially available software, Matlab, or the open-source software, Python together with SymPy package. On the other hand, if one can utilize highly accurate numerical methods, it is possible to develop software without being constrained by the above software. SoftCADS (Shiraishi et al., 2009) is one such software for calculating dynamic sensitivities with high accuracy, which achieves its accuracy through a combination of highly-accurate numerical differentiation methods (Shiraishi et al., 2007) and Taylor-series methods (Shiraishi et al., 2011). The sensitivity equations are automatically derived from the differential equations for metabolite concentrations and the sensitivities can always be obtained with reliable accuracy.

### Bottleneck ranking indicator (BR indicator)

When there is an enzyme reaction that strongly restricts the formation of a desired product in a reaction network (i.e., when there is a bottleneck enzyme), the productivity of the desired product may be augmented by increasing the activity of the bottleneck enzyme. Identification of the bottleneck enzyme is necessary for this purpose. The BR indicator expresses the degree of the “bottleneck” and can be used to identify the bottleneck enzyme (Sriyudthsak and Shiraishi, 2010a,b,c;Sriyudthsak et al., 2015). The BR indicator is calculated by:

(2) $$\begin{array}{r} L\left(X_{i}\left(t\right),Y_{j}\right)X_{i}\left(t\right)=\left(\frac{\partial X_{i}\left(t\right)}{\partial Y_{j}}\right)Y_{j}. \end{array}$$

Larger absolute values of the BR indicator indicate that the relevant enzyme indirectly restricts the formation of the desired product more strongly. The absolute values of BR indicators are ranked to determine which enzyme represents the bottleneck. The effectiveness of the BR indicator has been demonstrated in penicillin V and ethanol fermentation (Sriyudthsak and Shiraishi, 2010a,b,c). When microbial cells grow or a reactor is operated at non-steady state, the ranking of enzymes and therefore the designation of the bottleneck enzyme may vary with time (Sriyudthsak and Shiraishi, 2010a; Sriyudthsak et al., 2015).

### Network prediction

Metabolome data may also be used for the discovery of unknown metabolic pathways and their regulatory mechanisms. Rather than dealing with model construction, this deals with network analysis (Fukushima et al., 2014), which include correlation and causation networks. Metabolic profiles from different biological conditions can be used to build a correlation network, whereas the profiles of time series metabolic data can be utilized to generate a causation network. The correlation structure which is typically built by statistical methods using discrete data links the elements or nodes (i.e., metabolites) by their relations or edges (i.e., enzymatic reactions) of the metabolic networks. The correlation allows us to decrease the actual dimensionality of the system and qualitatively generate a probable network without comprehensive understanding of enzymatic reactions. For example, a system dynamics can be extracted from experimental time-series data using Takens theorem (Broomhead and King, 1986). In the case of large-scale metabolome data, the sample data obtained on different measurement scales can be processed using principal component analysis (Giuliani et al., 2004). Correlation network (Steuer et al., 2003) can also be visualized to understand relations among metabolites.

On the other hand, causation network links metabolites on the basis of their causal relationships. Two common approaches for generating a causal network are dynamic Bayesian network inference and Granger causality approaches for short and long length of time series data (Zou and Feng, 2009). More specific approaches, which combine mathematical and statistical approaches such as BST-loglem (Sriyudthsak et al., 2013a,b), were also proposed to predict metabolic pathways together with their regulatory mechanisms.

## Challenges and future perspectives

Regardless of past progress, there are still major challenges regarding the advantages and weaknesses of metabolomics integration and mathematical modeling. From our point of view, two major keystones that remain to be tackled are (1) the development of hybrid mathematical approaches for predicting a probable unknown pathway or regulatory mechanism from available metabolome data and (2) the integration of multilayer-omics data into large-scale kinetic models for designing optimal metabolic systems.

For the former issue (1), a challenge lies in the fact that there are thousands of metabolites inside of a cell, whereas metabolomics and mathematical modeling can usually handle hundreds of metabolites and it is, therefore, difficult to precisely predict which metabolite has an effect on a specific reaction pathway. A mathematical model in current approaches often gives several candidates for key regulatory metabolites, so that we still needs trial-and-error experiments in laboratory to narrow down the candidates on the basis of knowledge-based, but not data-driven, hypotheses. Thus, hybrid mathematical approaches combining existing and novel algorithms are expected to solve the issue.

For the latter issue (2), a significant challenge is the systematic integration of large-scale multilayer-omics data to understand metabolism not as a static system but as a dynamic one by capturing the dynamic behaviors and characteristics of all components inside a cell. Currently, it is possible to obtain metabolome, proteome, and transcriptome data. However, the systematic integration of those data by mathematical modeling approaches is still difficult. The selection of a suitable model type to deal with this circumstance has been long debated. A small-scale quantitative kinetic model can provide a detailed regulatory mechanism but only be applicable to a specific pathway. In contrast, a large-scale qualitative static model can offer the correlations among metabolites in a large-scale system but not clarify the detailed mechanism and dynamic behaviors. Thus, instead of selecting either approach exclusively, an improvement in a hybrid approach is desired for the construction of a large-scale kinetic model as well as the modeling of a multilayer-omics (or trans-omic) network, through the integration of the metabolome, proteome, and transcriptome (Yugi et al., 2014). It is anticipated that large-scale kinetic models for handling a trans-omic network would ultimately provide a holistic perspective with much greater insights into complex metabolic systems (Horgan and Kenny, 2011) to design and engineer the metabolism of a whole cell. An appropriated model will permit the precise prediction of dynamic behaviors and provide enormous possibilities for simulating entire cellular metabolisms, before practically exploiting the information to practical applications. Improved approaches to be established will pave the ways to computationally design an entire cellular metabolism and to simulate the behaviors, functions, and mechanisms of its components, like we can design a car and simulate the functions in each compartment.

## Author contributions

All authors listed, have made substantial, direct and intellectual contribution to the work, and approved it for publication.

## Funding

This research was supported in part by RIKEN Incentive Research Grant to KS, MEXT KAKENHI grant number 25119719 to FS, and JSPS KAKENHI grant number 25660084 to MYH.

### Conflict of interest statement

The authors declare that the research was conducted in the absence of any commercial or financial relationships that could be construed as a potential conflict of interest.
